# Pharmacological effects and target analysis of Guipi wan in the treatment of cerebral ischemia-reperfusion injury

**DOI:** 10.3389/fphar.2024.1346226

**Published:** 2024-03-07

**Authors:** Jianfeng Zhang, Li Luo, Yanyan Guo, An Liu, Mengjia Zhang, Wei Jiang, Xi Li, Qingqing Liu, Jiaoyan Yu

**Affiliations:** ^1^ Department of Pharmacy, Eighth Hospital of Xi’an City, Xi’an, China; ^2^ Department of Pharmacy, The Second Affiliated Hospital of Air Force Medical University, Xi’an, China; ^3^ Air Force Medical University, Xi’an, China

**Keywords:** guipi wan, ischemia-reperfusion injury, network analysis, GABBR1, PI3K/AKT

## Abstract

Guipi wan (GPW) is a traditional Chinese medicine commonly used in clinical practice, typically to treat neurological diseases such as neurasthenia and traumatic brain injury. It may have positive effects on cerebral ischemia‒reperfusion injury (cI/R). This study aimed to assess the effects of GPW in a mouse model of cI/R and find its possible targets. C57BL/6J mice were used to establish the cI/R model, and the laser speckle doppler was used to determine the success of the model. GPW was administered intragastrically for 7 days, brain tissue sections were stained with TTC, HE, and TUNEL, Western blot assay was performed to detect the effect of apoptosis-related proteins. Furthermore, we screened active ingredients from the TCM Database and constructed a compound‒target network using the Cytoscape 3.8.0 software. Moreover, we employed protein‒protein interaction and component‒target‒pathway network analyses to determine the potential components of GPW and its target genes, the key target was verified through molecular docking. Finally, we detected the influence of the downstream signaling pathway of the target through Western blot. The results showed that GPW decreased the cerebral infarction area, neurological function scores, and neuronal apoptosis in mice by regulating PI3K/AKT signaling pathway. Network analysis indicated that gamma-aminobutyric acid B receptor 1 (GABBR1) might be a potential target for the treatment of cI/R. Molecular docking indicated that 9 active components in GPW could bind to GABBR1 with desirable binding energy. This study represented the demonstratable effect of GPW in the treatment of cI/R injury and suggested GABBR1 as a potential target using network analysis.

## 1 Introduction

Ischemic stroke (IS) is the second leading cause of death worldwide and shows a high disability rate (Walter., 2022). It accounts for 70% of all strokes and carries a high risk of long-term recurrence. In 2019, the total number of IS-related deaths reached 3.29 million, accounting for 50.3% of stroke deaths and 17.7% of all cardiovascular disease-related deaths. And research predictive analysis suggested that this number could increase to 4.9 million by 2030 (Fan et al., 2023). Ischemic stroke occurs when blood flow to the brain is blocked due to a blocked or ruptured artery, disrupting the brain’s energy supply, causing tissue damage, and leading to widespread neuronal death (Tuo et al., 2022). The current treatment for this condition is the use of a recombinant tissue plasminogen activator, which triggers intravenous thrombolysis. However, the window for this treatment is narrow, and patients often face the risk of permanent disability after a stroke (Lin and Lang, 2022). In addition, blood flow restoration can cause more serious cerebral ischemia‒reperfusion injury (cI/R) (Campbell and Khatri, 2019). Therefore, a reliable treatment plan is urgently needed to improve the disability and nerve damage caused by reperfusion injury after a stroke.

To date, many drugs have been experimentally shown to have neuroprotective effects, but their clinical application has not yet been confirmed (Tsivgoulis et al., 2023). Specifically, traditional Chinese medicine (TCM) has been used to treat patients during the recovery period after a stroke, and many compound prescriptions have been demonstrated to be effective (Tao et al., 2020). Guipi wan (GPW) is often used to treat neurasthenia and traumatic brain injury, and also to improve the emotional and cognitive dysfunctions that arise after stroke (Li et al., 2020). It mainly comprises 11 traditional Chinese active ingredients, including Codonopsis radix, Atractylodes macrocephala rhizoma, Astragali radix, Glycyrrhizae radix et rhizoma, Poria, Polygalae radix, The seed of Ziziphus jujuba var. Spinos, Longan arillus, Angelicae sinensis radix, Aucklandiae radix and Jujubae fructus. Clinical experimental studies have confirmed that Astragali radix can significantly recover the clinical symptoms of cerebral ischemia, and play anti-atherosclerosis and neuroprotective roles. (Li et al., 2023a). Atractylenolide III in Atractylodes macrocephala Koidz ameliorates cerebral ischemic injury and neuroinflammation associated with inhibiting JAK2/STAT3/Drp1-dependent mitochondrial fission in microglia (Zhou et al., 2019). Several medicinal materials and components of GPW have been reported to improve cerebral ischemia reperfusion injury (Hao et al., 2023). These results suggest that GPW, as a clinical prescription of traditional Chinese medicine, may be effective in treating cerebral ischemia-reperfusion injury, but the underlying pharmacological mechanism remains unclear.

The systemic nature of TCM, which comprises multiple components with numerous downstream targets and complex mechanisms of action, cannot be accurately captured by the principles of Western medical research, which focuses on individual targets and components. Consequently, the Western approach has not been satisfactory in elucidating the pharmacodynamics of TCM compound prescriptions (Li et al., 2023b). However, with the advancement of network analysis techniques, more and more studies have begun to use this method to conduct holistic and systematic exploration to explain the mechanism of action of Chinese herbal medicine (Wang et al., 2021a), thus broadening their range of clinical applications. Many studies have successfully used network analysis to explain how effectively these drugs treat various diseases. Therefore, this study intends to combine pharmacological experiments and network analysis techniques to clarify the efficacy and possible targets of GPW in the treatment of cI/R.

This study confirmed that GPW has a significant therapeutic effect on cI/R model mice, in addition, predicted that GABBR1 is an important target for its therapeutic effect, and the downstream signaling pathway was verified, with the intent to elucidate elucidate the therapeutic effect and molecular mechanism of GPW on cI/R injury.

## 2 Materials and methods

### 2.1 Animals and drug administration

Adult male C57BL/6J mice aged 6–8 weeks, weighing 20–25 g, were provided by the Experimental Animal Center of the Fourth Military Medical University. The mice were housed at a constant temperature of 25°C ± 2°C and humidity of 50% ± 10%, with an alternating 12 h light/dark cycle and free access to food and water, they were randomly divided into five groups. Four groups underwent middle cerebral artery occlusion (MCAO) under anesthesia and then were administered a gastric injection with 0.9% saline or GPW extract (Lanzhou Foci Pharmaceutical, Lanzhou, 200,934, China).

GPW extraction process: Codonopsis radix (80 g), Atractylodes macrocephala rhizoma (60 g), Astragali radix (80 g), Glycyrrhizae radix et rhizoma (40 g), Poria (160 g), Polygalae radix (160 g). The seed of Ziziphus jujuba var. Spinosa (80 g), Longan arillus (160 g), Angelicae sinensis radix (160 g), Aucklandiae radix (40 g), Jujubae fructus (40 g), total 1,000 g. Codonopsis radix, Angelicae sinensis radix, Glycyrrhizae radix et rhizoma and Aucklandiae radix were crushed into fine powder, and the other medicinal materials 10 times the volume of water boiled 2 times, for 2 h each time, combined and concentrated into extract, and mixed with fine powder to make pill, weighing about 167 g, that is, 1,000 g of crude drug is equivalent to 167 g of extract, The quality of the extract was identified and the content of astragaloside in the extract detected by HPLC was 0.33 mg/g, which met the extraction requirement of ≥0.1 mg/g in Chinese Pharmacopoeia, as shown in Supplementary Figure S1. During gastric administration, 1 g extract was dissolved in 10 mL normal saline to obtain 6 g crude drug/1g extract/10 mL GPW administration solution.

The clinical dose of drug 15 g crude drug/60 kg used in humans was converted to approximately 3 g crude drug/kg in mouse, with a proportion of 12.3 times based on body surface areas (Reagan-Shaw et al., 2008), this dose was selected as the medium dose group. The low, medium and high doses calculated by the equal ratio of 2 times were 1.5, 3 and 6 crude drugs/kg. These values corresponded to the extract dosage of 0.25, 0.5, 1 g/kg, thereby obtaining the intragastric volume of 2.5 mL/kg, 5 mL/kg, and 10 mL/kg, respectively. The remaining group underwent a sham operation and then received intragastric administration of 0.9% normal saline.

### 2.2 MCAO model

The MCAO model was established with a modified suture method (Barthels and Das, 2020) After intraperitoneal injection of sodium pentobarbital anesthetized, the mouse was fixed in the supine position. Following alcohol disinfection of the neck skin, we incised the midline of the neck, bluntly separating the right common carotid artery (CCA), external carotid artery (ECA), and internal carotid artery (ICA). We threaded the CCA, ICA, and ECA separately. Next, we clamped the proximal end of the CCA and the ICA, and tied the two cords at the proximal end of the ECA to a dead knot, while we tied the cords at the proximal end of the ECA to a slipknot. We cut a small “V" between the two cords at the proximal end of the ECA, inserted the thread plug, and insert the thread. We tightened the proximal end of the ECA line, fixed the line tether, lifted the ECA, and untied the ICA arterial clamp. We then slowly pushed the line tether toward the ICA. When the thread end entered about 1.2 cm, we stopped when we felt some resistance. We then untied the CCA artery clip, at this time. The end of the thread plug was just at the beginning of the middle cerebral artery (MCA), causing an occlusion. After 2 h of ischemia, the thread plug was pulled out to generate the ischemia‒reperfusion model.

### 2.3 Laser speckle Doppler

Mice were anesthetized by intraperitoneal injection of 1% sodium pentobarbital solution at 50 μg/kg of body weight. Once anesthetized, the head was fixed on the operating table, the skin was cut along the midline, connective tissue was removed, and brain blood flow images were collected by laser speckle doppler flow imager (RFLSI 111).

### 2.4 Neurological scores

After model establishment and drug or saline administration, neurological deficits were assessed with a 5 point scoring system, as previously described (Kuai et al., 2021). According to this method, a score of 0–4 corresponds to no neurological dysfunction, inability to fully extend the left front paw, hovering left, falling left, low level of consciousness and inability to walk autonomously. A score of 5 represents death.

### 2.5 2,3,5-Triphenyltetrazolium chloride staining

After 24 h, the mice in each group were deeply anesthetized, their brain tissue was removed, and residual blood was washed out with normal saline. Brains were then quickly frozen at −20°C for 20 min and cut into 2 mm slices with a brain slice mold (68,714, RWD Life Science). Sections were stained with 1% 2,3,5-triphenyltetrazolium chloride (TTC, purity >98.0%; Sigma-Aldrich, St Louis, MO, United States) for 30 min at 37°C. ImageJ software (NIH) was used to measure the percentage of tissue affected by cerebral infarction.

### 2.6 Hematoxylin and eosin staining

Brain tissue was immersed in 4% paraformaldehyde for 24 h and then embedded in paraffin. Thereafter, 4-μm-thick sections were prepared for hematoxylin and eosin (HE) staining. After dehydration with a gradient series of ethanol and xylene, the brain tissue structure was observed under an optical microscope (Ts2-FL, Nikon, Tokyo, Japan).

### 2.7 Nissl staining

Paraffin sections were sequentially dehydrated with xylene, and absolute, 95%, 80%, and 70% ethanol. Sections were then washed with distilled water, soaked in 1% tar violet dye for 10 min, washed with distilled water again, and decolorized with gradient ethanol immersion. After becoming transparent, sections were mounted on clear slides with neutral gum, and tissue was observed under an optical microscope.

### 2.8 TUNEL staining

Sections were washed twice for 5 min with xylene. After gradient ethanol immersion, sections were infused with proteinase K to increase cell permeability and incubated at 37°C for 20 min. Next, sections were first incubated with 100 μL of TUNEL (6-Diamidino-2-phenylindole) reaction mixture (ab66110, Abcam) at 37°C for 1 h under dark conditions, and then with 100 μL DAB solution at room temperature for 10 min. Counterstaining with hematoxylin was performed for 3 min and, after rinsing, sections were dehydrated in a graded series of ethanol, made transparent in xylene, and mounted on neutral gum. The number of TUNEL-positive cells was counted under a fluorescence microscope (Ts2-FL, Nikon), and the percentage of TUNEL-positive cells was calculated to calculate the rate of apoptosis.

### 2.9 Western blotting

For Western blotting, 20 mg of brain tissue from the infarct area was lysed in 100 μL RIPA buffer, and homogenized by ultrasonication. Samples were centrifuged at 12,000 rpm for 10 min at 4 °C. The supernatant was removed, and the total protein concentration in each sample was quantified by bicinchoninic acid assay (Pierce BCA Protein Assay Kit; Thermo Fisher Scientific, Waltham, MA, United States). After heating the samples in the presence of a loading buffer, SDS-PAGE (sodium dodecyl sulphate-polyacrylamide gel electrophoresis) was performed on 8–10% gels. Proteins were later transferred to PVDF membranes, which were blocked in 5% skim milk for 1 h and incubated at 4°C overnight with primary antibodies (1:1,000 dilution) for PI3K (13666S; Cell Signaling Technology, Danvers, MA, United States), AKT (2920ST, Cell Signaling Technology), P-AKT (13038S, Cell Signaling Technology), caspase-3 (9664T, Cell Signaling Technology), cleaved caspase-3 (9664s, Cell Signaling Technology), Bcl-2 (3498s, Cell Signaling Technology), Bax (14796s, Cell Signaling Technology), and β-actin (059M4770v, Sigma-Aldrich). After repeated washing, membranes were incubated with secondary antibodies (1:5,000 dilution) at room temperature for 1 h and subsequently washed. Membranes were then observed and photographed through a gel imager (Bio-Rad, United States). ImageJ was utilized to analyze the relative expression levels of the proteins, with β-actin as a reference.

### 2.10 Target prediction of GPW components

We used the Traditional Chinese Medicine System Pharmacological Analysis Platform (TCMSP, http://tcmspw.com/tcmsp.php) and the Bioinformatics Analysis Tool for Molecular mechanism of Traditional Chinese Medicine (BATMAN-TCM) database (http://bionet.ncpsb.org/batman-tcm) to identify the main active compounds of GPW. The screening conditions were the following: oral bioavailability (OB) ≥ 30%, drug-like activity (DL) ≥ 0.1, and blood‒brain barrier (BBB) ≥ 1 (Liu et al., 2020). The information concerning the main active compounds of GPW and their corresponding targets was collected and standardized through the Uniport database (https://www.uniprot.org/), and the gene names of each target were obtained.

### 2.11 Compound-target network construction

Cytoscape 3.8.0 (National Institute of General Medical Sciences, Bethesda, MD, United States) was used to construct a compound-target network for GPW. We also used the GeneCards (https://www.genecards.org/), OMIM (https://omim.org) and DisGeNET (https://www.disgenet.org/) databases with cI/R as the keyword to acquire the key therapeutic targets for cI/R. The downstream targets of GPW and cI/R were imported into the online platform of Venny 2.1.0 for analysis, and Venn diagrams were used to visualize the common targets of GPW and cI/R injury.

### 2.12 Protein-protein interaction network analysis

The common targets were imported into the STRING database (https://string-db.org/), and “multiple proteins” and “*Homo sapiens*” were selected to obtain protein-protein interaction (PPI) data. This information was imported into Cytoscape 3.8.0 to perform a visual analysis by drawing PPI network diagrams. The CytoHubba plug-in was employed for network topology analysis (Athanasios et al., 2017). We selected targets with a degree higher than the average as the key targets in the PPI network.

### 2.13 Gene function annotation and pathway enrichment analysis

To study the potential molecular mechanism through which GPW exerts positive effects on cI/R, the key targets were analyzed by gene function annotation (Gene Ontology, GO), and pathway enrichment analysis (Kyoto Encyclopedia of Genes and Genomes, KEGG). Statistical significance was set at *p* < 0.05. Finally, Cytoscape 3.8.0 was employed to construct a component‒target‒path (CTP) network to study the reciprocity of components, targets, and paths (Cui et al., 2020). In such a network, the degree value of a node was defined as the number of edges connected to the rest of the network, where each edge corresponds to the interaction between a biologically active ingredient and its target, thus representing the physiological relevance of the node in the network.

### 2.14 Molecular docking studies

The main chemical components and the core targets obtained from database screening were verified by molecular docking analyses (Taha et al., 2020). The structures of the key components were searched on the PubChem database (http://zinc. docking. org/), optimized, and saved in. mol format (Xia et al., 2020). The Cryo-EM structure of GABBR1 was downloaded from the Protein Data Bank (PDB) and saved in. pdb format. Water molecules and the docking ligand were removed with the PyMOL software (Schrödinger, New York, NY, United States). The obtained protein structures were then imported into AutoDock (Center for Computation Structural Biology, The Scripps Research Institute, La Jolla, CA, United States) to apply pre treatments such as hydrogenation. The active ingredients of GPW and the target proteins were saved as. pdbqt files, and AutoDock was utilized to simulate the docking of each active ingredient and target protein. The data concerning the lowest binding energy resulting from molecular docking was obtained. The lower the binding energy, the stronger the binding force between the active ingredient and the target protein: a drug molecule with binding energy ≤ −5.0 kJ/mol is considered to have a good binding activity to the target (Li et al., 2021).

### 2.15 Statistical analysis

All data are expressed as mean ± standard deviation (Mean ± SD). SPSS19.0 (IBM, Armonk, NY, United States) and GraphPad 9.0 (GraphPad, La Jolla, CA, United States) were used for statistical analysis and graph plotting. *t*-test was used to compare the means of two samples. A one-way analysis of variance and Tukey’s test were employed for comparisons of three or more group means. Statistical significance was set at *p* < 0.05.

## 3 Results

### 3.1 GPW reduced the cerebral infarction area in MCAO mice

**FIGURE 1:**
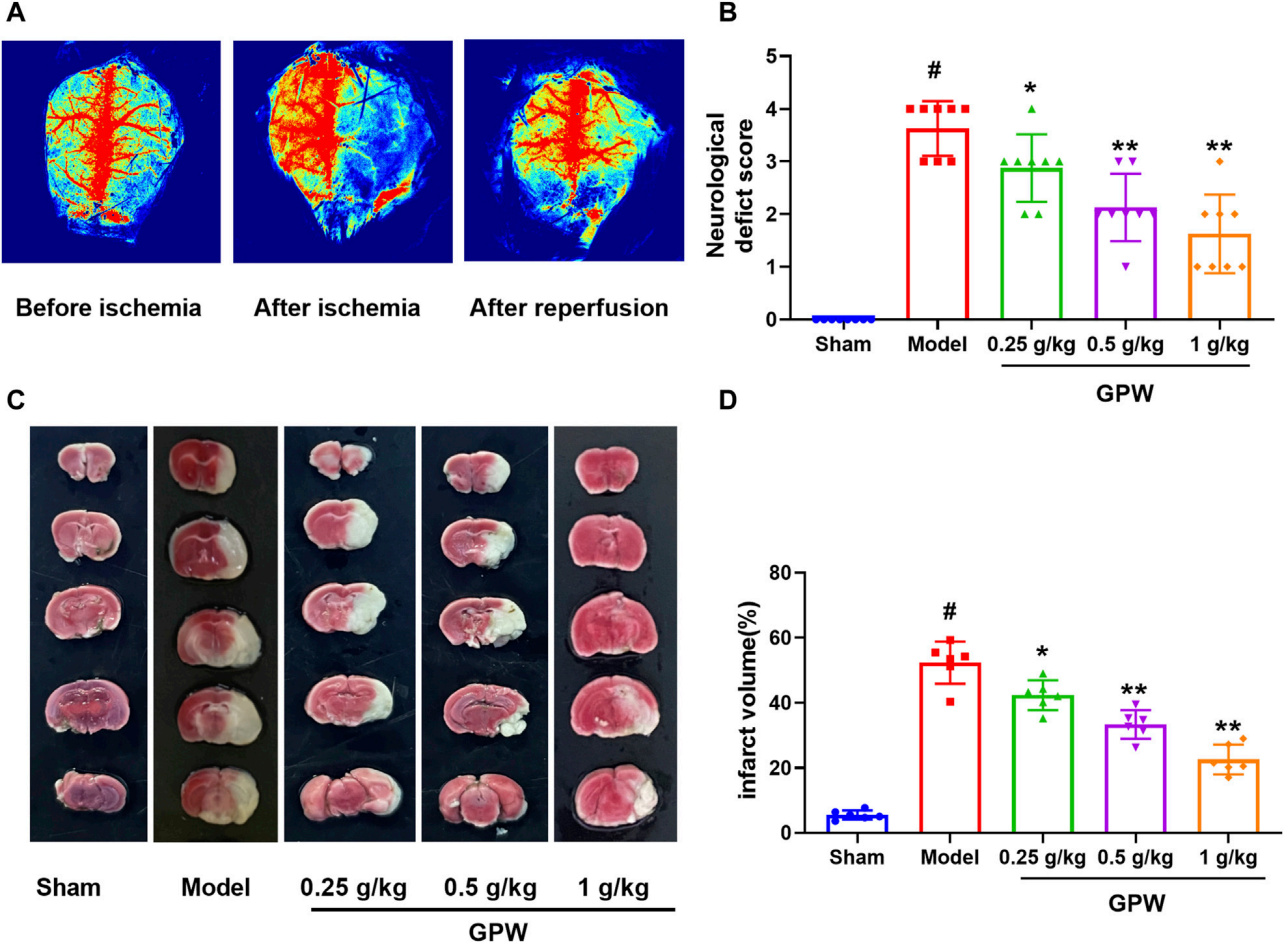
GPW reduces the brain damage caused by cerebral ischemia/reperfusion injury (cI/R). **(A)** Laser speckle Doppler shows the success of cI/R induction. **(B)** Neurological function score. *N* = 8 **(C)** Representative images of TTC staining in brain sections from different experimental groups. **(D)** Quantitative assessments of the infarct area from TTC staining. *N* = 6; ^#^ *p* < 0.01 *versus* the sham group; *p < 0.05, **p < 0.01 compared with the Model group.

The total blood flow on the ischemic side after reperfusion was measured by laser speckle Doppler, and blood flow recovery was greater than 70%, indicating successful induction of cI/R (Figure 1A). Different doses of GPW significantly improved the neurological score of the model mice (Figure 1B). TTC staining showed that GPW significantly reduced the size and volume of the infarct in the model mice compared with that in the sham operation group (Figures 1C, D).

### 3.2 GPW reduced neuronal damage in MCAO mice

**FIGURE 2:**
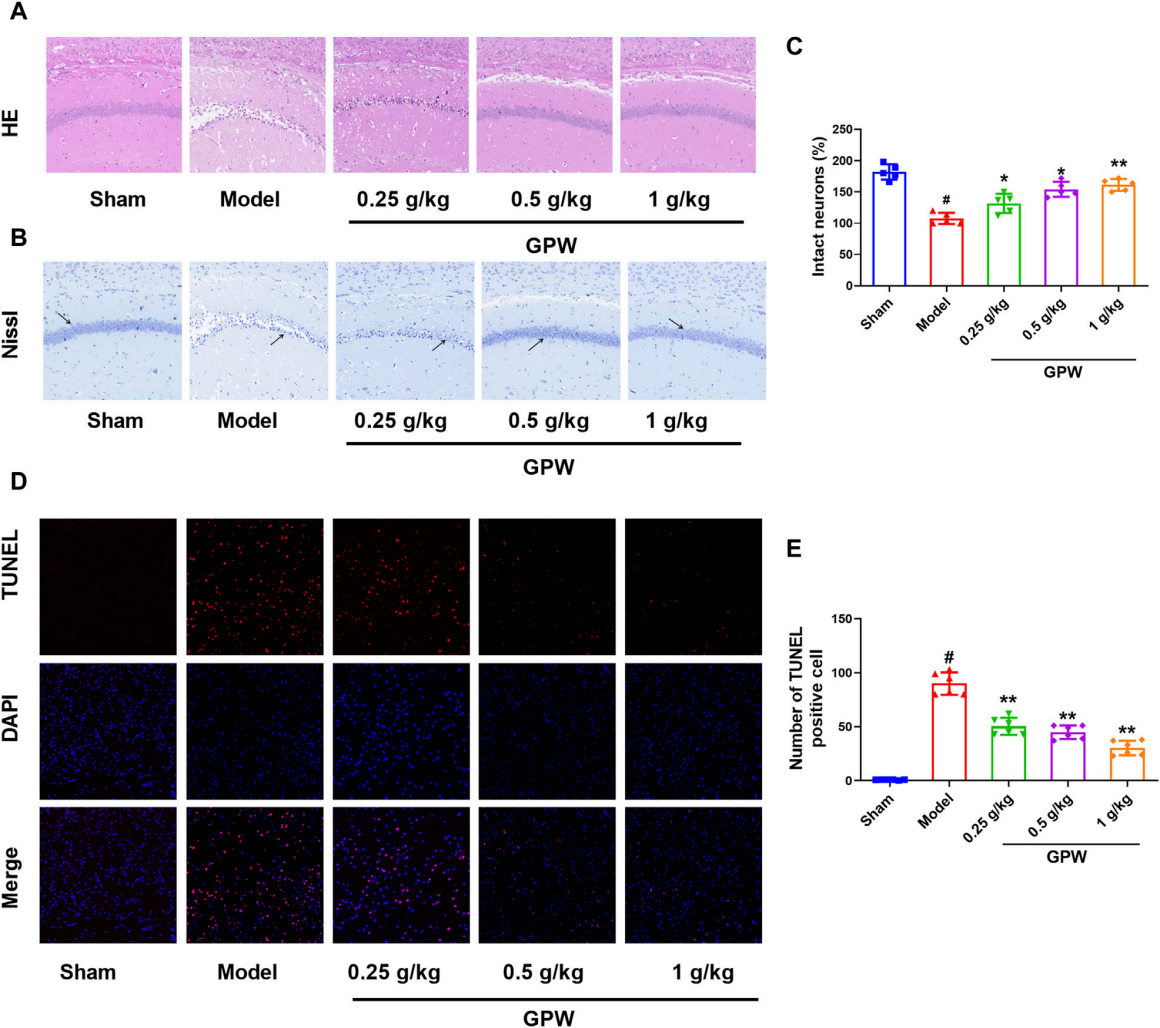
GPW alleviates the pathological damage of model mice. **(A)** Hematoxylin and eosin staining results of infarct area (20×). **(B)** Nissl staining results of infarct area (20×), The black arrow shows the Nissl corpuscles. **(C)** Numerical analysis of Nissl staining with ImageJ program. **(D)** TUNEL staining results of brain tissue sections in different experimental groups. **(E)** Statistical analysis of the percentage of intact neurons in the infarct area of mice by TUNEL staining. Scale bar = 75 μm; *N* = 6 mice per group; ^#^ *p* < 0.01 *versus* the sham group; *p < 0.05, **p < 0.01 compared with the Model group.

HE staining showed that, in the sham group, hippocampal neuronal cell bodies were large and round with obvious nucleoli, and the cells were arranged in multiple well-organized layers. In the model group, the number of cells on the affected side was reduced, the arrangement was sparse, the cell bodies were shrunken, the nuclei were constricted in triangles, and some cell nuclei had disappeared. On the contrary, the proportion of atrophied neurons in the GPW-treated groups was significantly reduced (Figure 2A). Nissl staining showed the same trend. Compared with the sham group, the number of Nissl bodies in the model group was significantly reduced, while that in GPW-treated model groups was significantly increased (Figures 2B, C).

Furthermore, almost no TUNEL-positive cells were observed in the sham group, but their number was significantly increased in the model group. After GPW administration, the number of TUNEL-positive cells in the model mice was reduced in a dose-dependent manner (Figures 2D, E). This further revealed that GPW could reduce neuronal apoptosis of cI/R injury.

### 3.3 GPW decreased the expression of apoptosis-related proteins in MCAO mice

**FIGURE 3:**
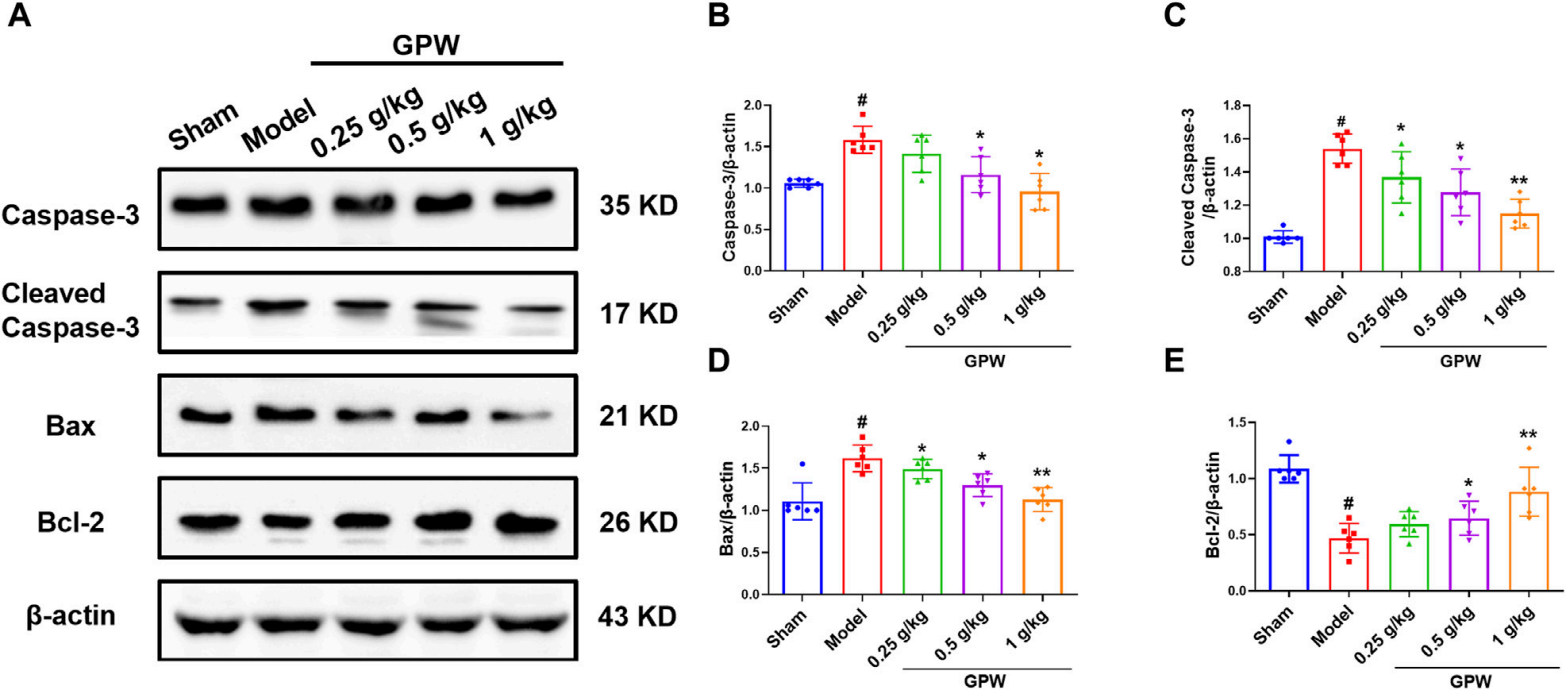
Effect of GPW on apoptosis-related proteins. **(A)** Western blot detection of caspase 3, cleaved-caspase 3, Bax, and Bcl-2 bands in the brain tissue of each group of mice. **(B**–**E)** Statistical analysis of protein expression. *N* = 6 mice per group; ^#^ *p* < 0.01 *versus* the sham group; *p < 0.05, **p < 0.01 compared with the Model group.

The expression of the pro-apoptotic proteins caspase-3, cleaved caspase-3, and Bax was significantly increased in the model group but significantly decreased after GPW administration (Figure 3A‒D). Accordingly, the expression of the anti-apoptotic protein Bcl-2 was significantly reduced in the model group, while the administration of GPW was able to counteract this decrease (Figure 3E). These results indicated that GPW had the potential to inhibit neuronal apoptosis at the molecular level.

### 3.4 Network analysis predicted the main related targets of GPW

**FIGURE 4:**
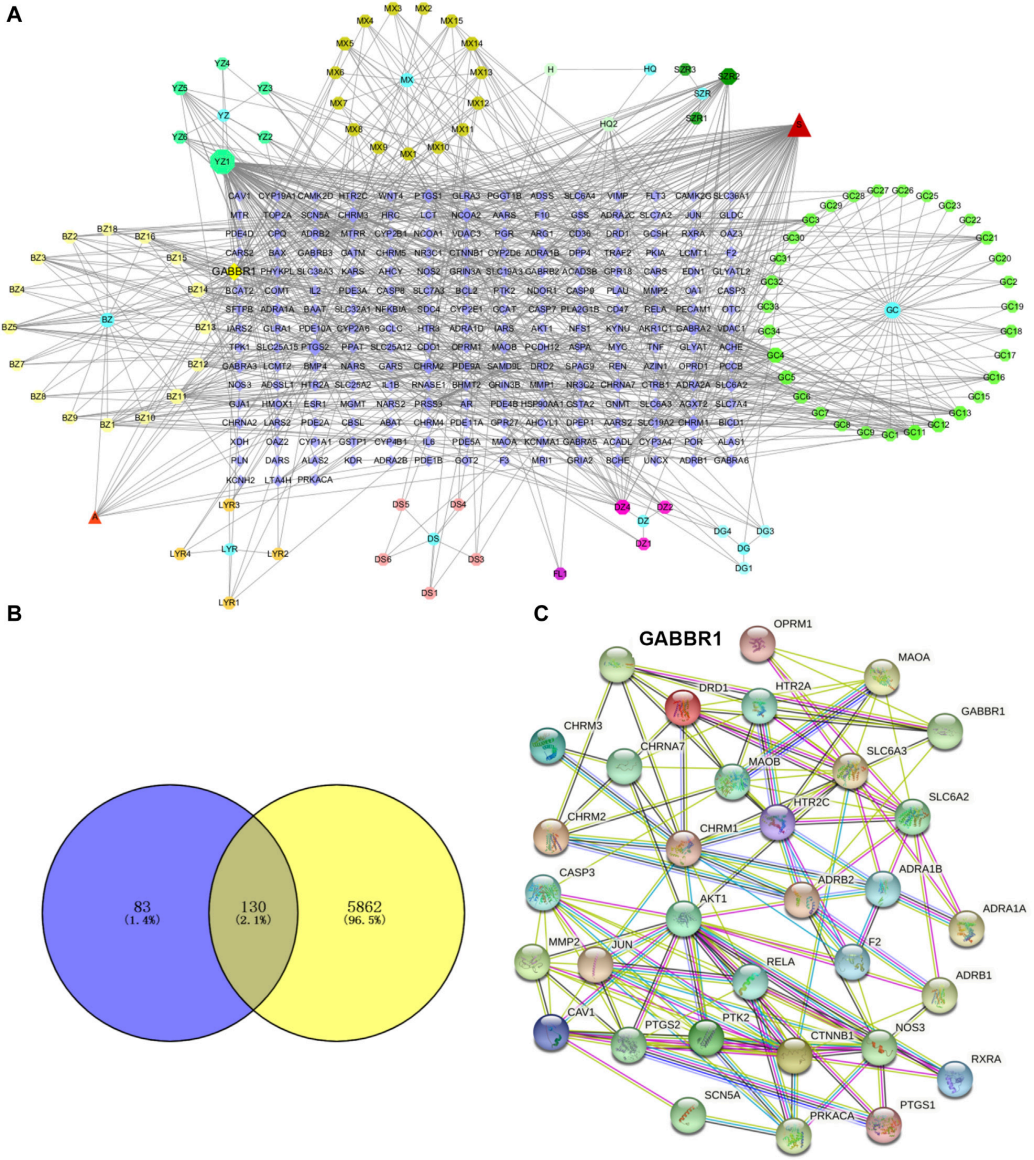
GPW component selection and target prediction. **(A)** The active ingredients of GPW and their targets are represented by nodes. The circles represent the plant components of GPW, the number of the corresponding compound is found in Supplementary Table S1, the polygons of different colors represent the main components of each plant, and the purple square represents the relevant target of the component. Edges represent the interaction between the biologically active ingredient and the target. **(B)** The Venn diagram of GPW and cI/R targets. Purple represents component targets, yellow represents disease targets, and the intersection represents common targets. **(C)** Protein‒protein interaction network diagram. Nodes represent different proteins, and lines represent interactions.

The role of GPW in the treatment of cI/R has been determined; further, it is intended to predict its possible targets through network analysis. Through TCMSP, according to the conditions of OB ≥ 30%, DL ≥ 0.1, and BBB ≥1, 95 active components were selected from 11 herbs (Supplementary Table S1). Using the uniprot databases to normalize cI/R targets and delete duplicates, a total of 213 targets were obtained. Then we used cystoscope software to construct the “component-target network” diagram of GPW (Figure 4A). In this diagram, the “degree” is defined as the number of connected edges in the network, representing the importance of the network. We found a total of 29 compounds with a degree value greater than the average value of 8.76. Among these, we further excluded 12 compounds due to a lack of compatibility with Lipinski’s rules (Chen et al., 2020); 2) the structure conforming to the characteristics of Pan-Assay Interference Compounds (PAINS), which could indicate positive readouts in biochemical assays via different mechanisms (Grigalunas et al., 2020). Thus, only 17 compounds were selected for the prediction of subsequent targets (Table 1).

**TABLE 1:** Compounds screened according to degree value and druggability.

Molecule name	Degree	Molecule name	Degree
Nuciferine	31	Selina-4(14),7(11)-dien-8-one	11
Atractylone	19	(−)-Caryophyllene oxide	11
Beta-carotene	19	5,6,7,8-Tetrahydro-2,4-dimethylquinoline	11
3β-Acetoxyatractylone	17		
Benzo [a]carbazole	16	Juniper camphor	10
Coumarin	14	Harman	10
Harmine	13	Ermanthin	9
(+/−)-Isoborneol	13	2-[(2R,5S,6S)-6,10-dimethylspiro [4.5]dec-9-en-2-yl]propan-2-ol	9
α-Cubebol		13	
(5E,9Z)-3,6,10-trimethyl-4,7,8,11-tetrahydrocyclodeca [b]furan		11	

**FIGURE 5:**
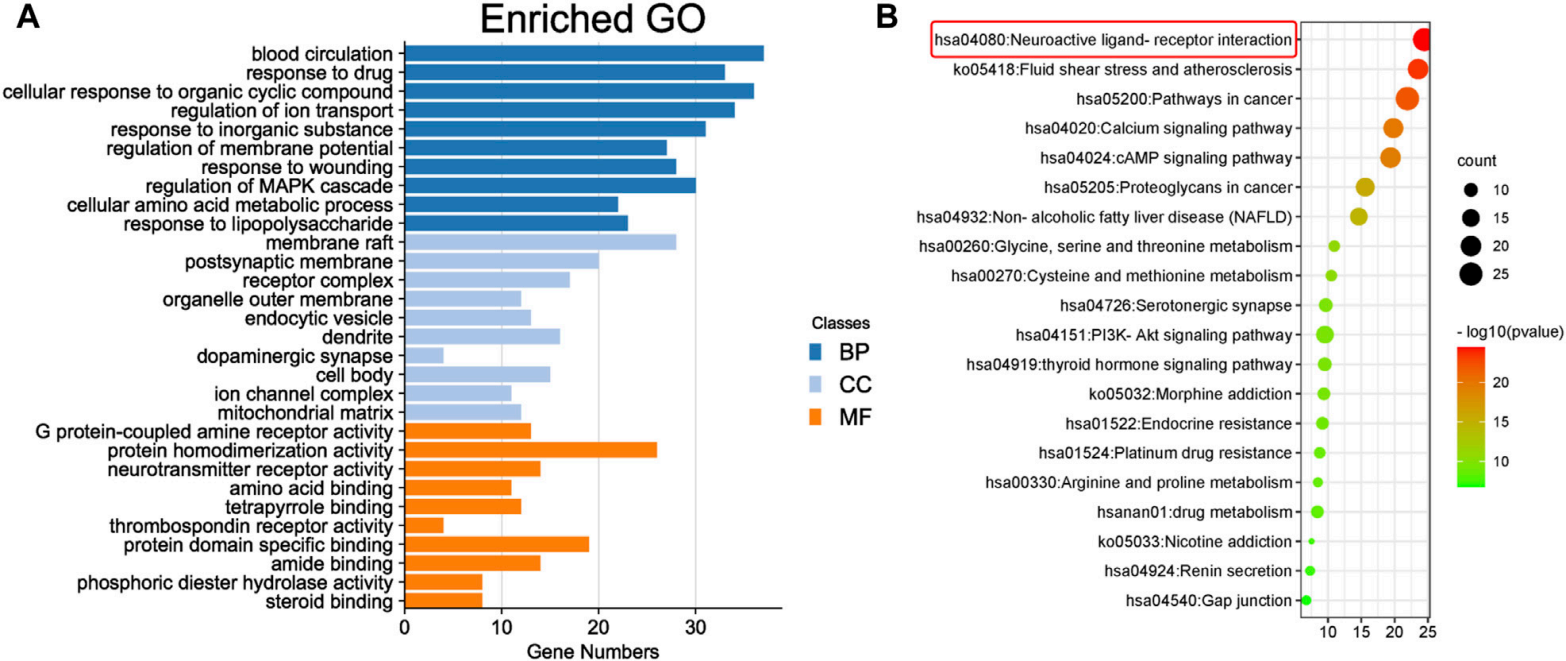
Enrichment analysis of core targets. **(A)** The top 10 results for the biological process (BP), cell component (CC), and molecular function (MF) categories in Gene Ontology (GO) annotation analysis. The *y*-axis represents the target-enriched GO, and the *x*-axis represents the gene number in the GO annotation results. **(B)** Top 20 Kyoto Encyclopedia of Genes and Genomes (KEGG) pathway enrichment bubble chart (*p* < 0.05). The *y*-axis represents the name of the enriched pathway, and the *x*-axis represents the enrichment value.

To predict the target receptor of GPW for the treatment of cI/R, we performed the analysis from three aspects. First, we list the top 20 targets among the 213 possible action targets selected based on the degree values (Table 2) and found theγ-aminobutyric acid receptor (GABBR1) with the degree value of 38. Second, we selected 130 target receptors that are relevant to the treatment of cI/R with GPW from 5,992 (Figure 4B). After drawing the PPI network structure with the STRING database website, 36 targets with degree values greater than the average were obtained for visualization (Figure 4C). The results showed GABBR1 is included in the main genes that can be exploited for treatment of cI/R. Finally, we employed the GO and KEGG pathway enrichment combined with CTP network analysis. As illustrated in Figure 5A, the top 10 biological process (BP), molecular function (MF), and cellular composition (CC) were represented from 130 potential targets. Furthermore, KEGG analysis revealed that 112 signal pathways are involved in GPW-mediated effects on cI/R injury, among which the top 20 were selected to make a bubble map (Figure 5B). According to the results, GPW mainly acts on cI/R injury through neuroactive ligand‒receptor interaction, fluid shear stress, atherosclerosis, calcium signaling pathway, and the cAMP and PI3K/AKT signaling pathways. Interestingly, GABBR1 is a key target in the neuroactive ligand-receptor interaction signaling pathway. Thus, these results, taking three aspects of drug intervention, disease correlation, and pathway enrichment, demonstrated GABBR1 may be an important target for GPW treatment of cI/R.

**TABLE 2:** The top 20 targets out of 213 possible GPW targets.

Target name	Degree	Target name	Degree
GABBR1	38	SLC6A2	15
PTGS2	30	ADRB2	13
CHRM1	29	ADRA1A	13
CHRM2	29	GABRA3	13
CHRM3	28	BCHE	12
NCOA2	21	GABRA6	12
GABRA2	20	ADRA1B	13
PTGS1	18	SCN5A	10
PRSS3	16	SLC6A3	10
CHRNA7	16	RXRA	10

### 3.5 Molecular docking further confirmed that GABBR1 might be a reliable target for GPW therapy cI/R

**FIGURE 6:**
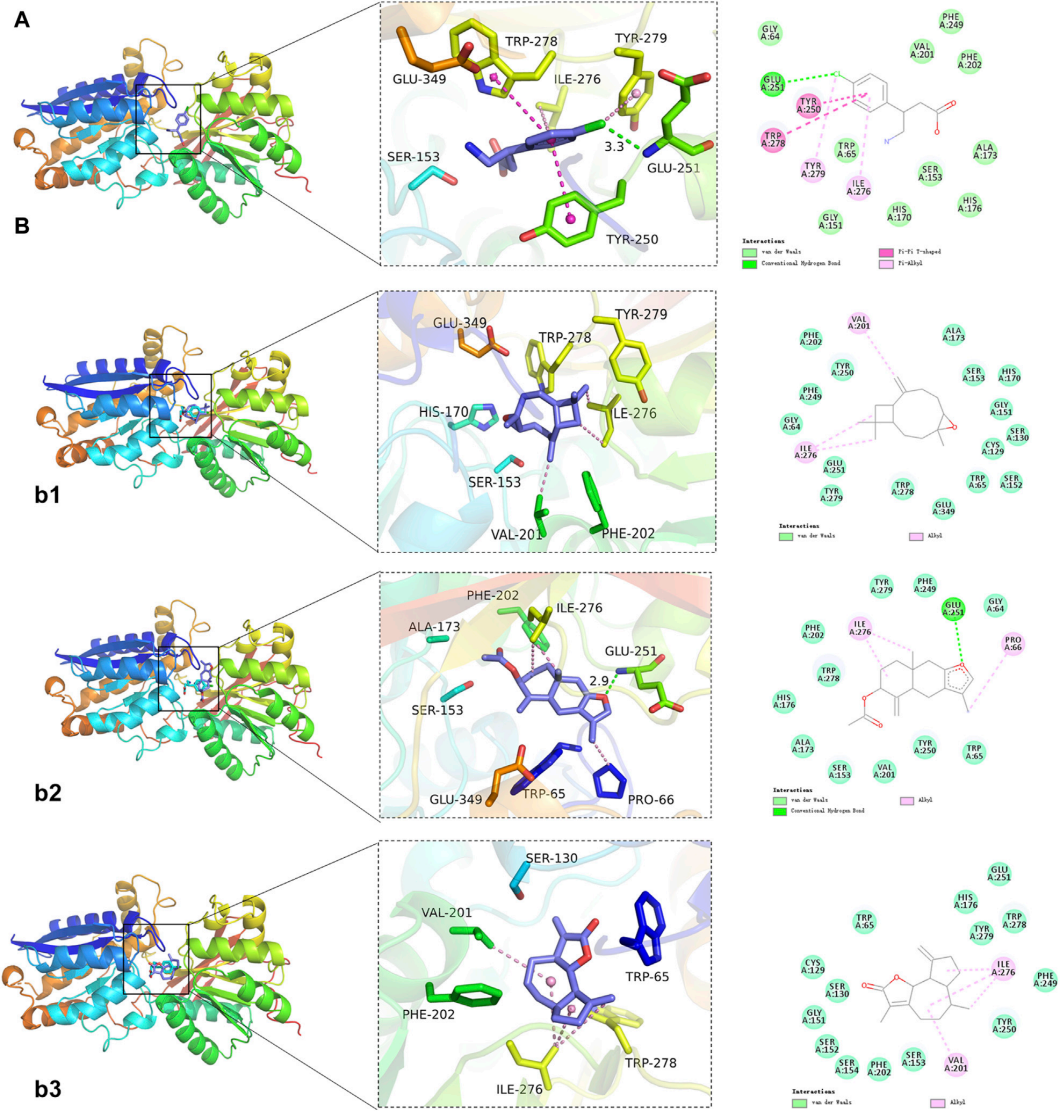
Representative results of molecular docking analyses. **(A)** Binding of the positive control baclofen to GABBR1. **(B)** Representative docking results of 3 GPW components. b1: (−)-Caryophylleneoxide; b2: 3β-acetoxyatractylone; b3: Ermanthin.

To further explore the binding mechanism of GPW on cI/R, we obtained 8 compounds including 3β-acetoxyatractylone, (+/−)-isoborneol, α-cubebol, selina-4(14),7(11)-dien-8-one, (−)-caryophyllene oxide, juniper camphor, 2-[(2R,5S, 6S)-6,10-dimethylspiro [4.5]dec-9-en-2-yl]propan-2-ol, and ermanthin after intersecting the 17 main components of the preliminary screening with 38 components that interact with GABBR1 (Supplementary Figure S2A). Here, we intend to explore the binding mechanism between these active compounds and GABBR1. As illustrated in Figure 6A, taking baclofen as a probe, we observed baclofen formed one hydrogen bond with Glu251, the alkyl conjugation interactions with Tyr279, and Ile276, and π-π interactions with Tyr250 and Trp278. In addition, the formation of the ligand-receptor complex was also dependent on van der Waals forces with Ser153, Ser131, Trp65, Tyr279, and Phe202. The binding energy of baclofen was −7.0 kcal/mol, indicating the strong binding ability between baclofen and GABBR1.

Next, we performed the molecular docking between these 8 compounds and GABBR1. As illustrated in Figure 6B; Supplementary Figure S2B, we obtained the binding energies ranked −6.3 to −3.4 kcal/mol (Table 3), whereby suggested that these 8 compounds had good binding ability with GABBR1. Similarly, the formation of these 8 ligand-receptor complexes was also based on the hydrogen bond, conjugation interactions, and van der Waals forces formed by 8 compounds with the above-mentioned key amino acid residues in GABBR1. In parallel, these results indicated that the mechanism of the interaction between bioactive compound and GABBR1 was similar to that of clinical drugs, thus proving the promising potential of GPW for cI/R treatment.

**TABLE 3:** Molecular docking results between ligands and core target receptors.

Molecule name	Binding energy (kcal/mol)
Baclofen (GABBR1 agonist)PTGS2	−7.0
3β-acetoxyatractylone	−5.3
(+/−)-Isoborneol	−4.2
α-Cubebol	−5.1
Selina-4(14),7(11)-dien-8-one	−3.4
(−)-Caryophyllene oxide	−6.3
Juniper camphor	−4.8
2-[(2R,5S,6S)-6,10-dimethylspiro [4.5]dec-9-en-2-yl]propan-2-ol	−5.7
Ermanthin	−5.8

### 3.6 GPW affected the expression of PI3K/AKT signaling pathway-related proteins

**FIGURE 7:**
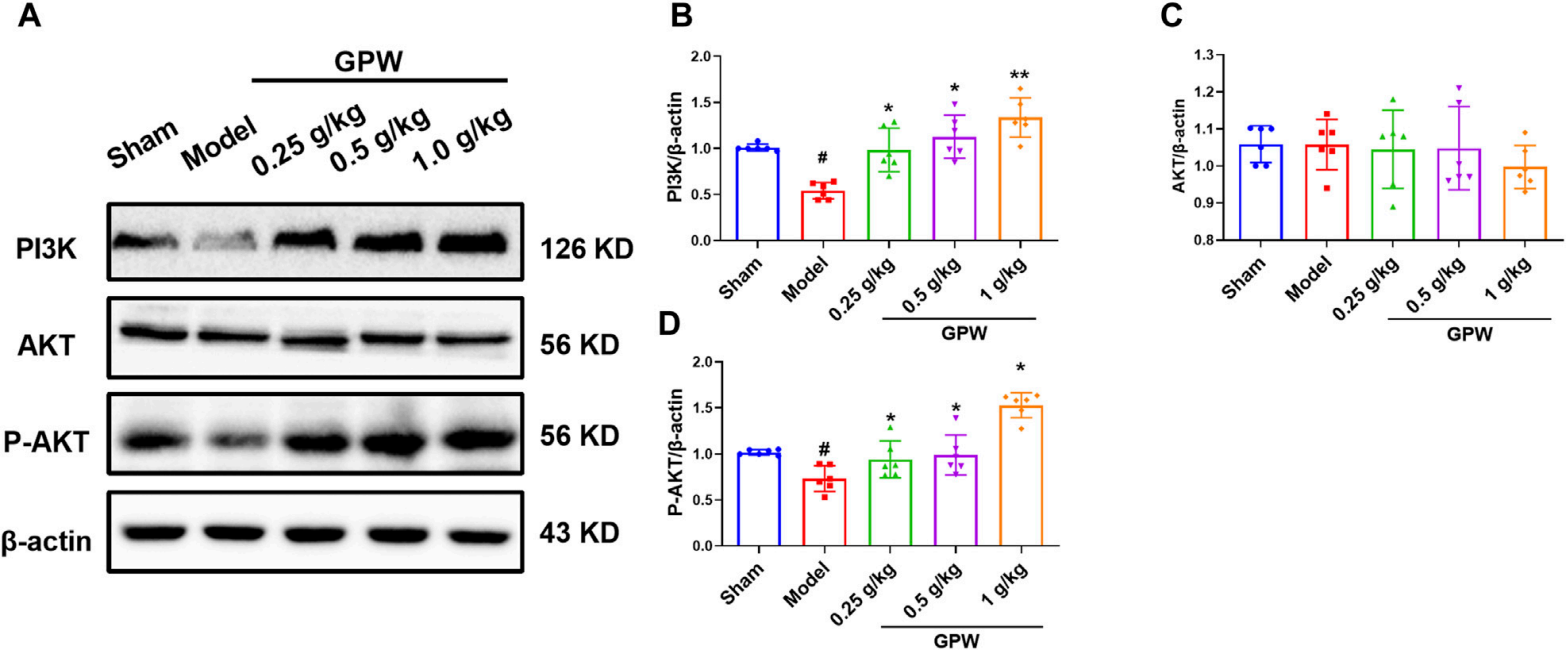
Effect of GPW on PI3K/AKT signaling pathways in the infarct area. **(A)** WB representative result chart. **(B)** Statistical analysis results of PI3K expression. **(C)** Quantitative statistical results of AKT expression. **(D)** Quantitative statistical results of the p-AKT expression. *N* = 6 mice per group; ^#^ *p* < 0.01 *versus* the sham group; *p < 0.05, **p < 0.01 compared with the Model group.

It has been shown that modulating GABBR1 can inhibit apoptosis of rat hippocampal neurons through the PI3K/Akt pathway in the treatment of refractory epilepsy. We speculate that GPW may affect the PI3K/AKT signaling pathway by activating GABBR1, Therefore, We used Western blotting to evaluate changes in the expression of the related proteins (Figure 7A). Compared with the sham group, the model group had significantly reduced levels of PI3K, whose expression increased after GPW administration (Figure 7B). Also, while the total expression of AKT did not change (Figure 7C), that of phosphorylated AKT (p-AKT) was significantly reduced in the model group; this reduction was restored in GPW-treated mice (Figure 7D). In summary, the results show that GPW can activate the PI3K/AKT signaling pathway.

## 4 Discussion

In this study, we validated the pharmacological effect of GPW in the treatment of cI/R injury. The prevention and treatment of ischemic stroke remain a worldwide challenge. A central issue is the risk of bleeding associated with anticoagulants and antiplatelet drugs (Sandercock et al., 2015). Due to its low side effects, TCM is often sought as an alternative drug therapy for ischemic stroke prevention and rehabilitation intervention in China. Natural medicines in TCM are the origin of many new medicines, such as Salvia miltiorrhiza, which has been used to treat cerebrovascular diseases in China for thousands of years. TCM has been used in humans for more than 2,000 years. The valuable experience provided by this practice can provide powerful guidance for drug discovery (Sun et al., 2015). However, there are still some limitations of TCM, such as the low quality of TCM tests and unclear ingredients and mechanisms. GPW in this study is a classic prescription in TCM, which has been reported to be effective against many neurological diseases. However, its effect and mechanism of action on cI/R injury have not been reported. This study is of great significance for the development of therapeutic drugs for ischemic stroke based on TCM.

Firstly, we used the classical mouse MCAO model to further evaluate the therapeutic effect of GPW (Walter, 2022). The success of the model was verified by laser speckle Doppler, and GPW was found to reduce cerebral infarction size and neural function score and reduce neuronal apoptosis in model mice. Apoptosis is an important pathway leading to neuronal death after stroke, and caspase-3, bax, and blc-2 are apoptosis-related proteins. We further used WB to find that GPW can counteract these changes in stroke levels. The effect of GPW on cI/R was confirmed by experimental pharmacological methods.

Next, the main components and targets of cI/R treatment with GPW were screened and predicted by network analysis. Using the TCMSP database and according to OB, DL, and BBB standards, 96 components of 11 kinds of Chinese herbs in GPW were screened out. Further, the degree values were ranked according to the number of corresponding targets, those less than the average are excluded, and those greater than the average are 29 in total. The druggability of the compound requires compliance with Lipinski’s Rule of Five and the PAIN rule, which further excludes some compounds, S-(2-Carboxyethyl)-L-Cysteine and Stigmasterol meet the druggability rules, but there are many targets and poor specificity. Therefore, 17 compounds such as nuciferine, atractylone, β-carotene, and coumarin were obtained. Among these, previous studies have shown that nuciferine was found to significantly improve neurological deficit scores, cerebral edema, and infarction by regulating fat metabolism and inflammatory response (Wu et al., 2020). Similarly, patients with higher serum β-carotene were found to have a significantly lower risk of stroke and death (Huang et al., 2018). Additionally, coumarin is a common oral anticoagulant used for the prevention and treatment of stroke (Cordonnier, 2018). These findings, together with our results, confirm that the core components of GPW can effectively reduce cI/R injury. However, although atractylone, 3β-acetoxyatractylone, and harmine have not been reported to have a therapeutic effect on ischemic stroke, they may play a synergistic role in the treatment of GPW, which needs to be verified by subsequent experiments.

Furthermore, in the results of network analysis, GABBR1 was found to have the highest degree in the target of GPW components. Additionally, combined with the analysis results of PPI, GO, and KEEG analysis found that GABBR1 was closely related to the effect of GPW on cI/R. Combined with the above results, we analyzed and speculated that GABBR1 may be a key target for the treatment of cI/R by GPW. Molecular docking provides further validation and suggests that multiple components in GPW may synergistically act on GABBR1 to produce neuroprotective effects. GABBR1 is the metabolic receptor 1 subtype of the inhibitory neurotransmitter gamma-aminobutyric acid (GABA), It is a G protein-coupled receptor expressed in neurons and glial cells throughout the brain (Cediel et al., 2022). GABBR1 exerts presynaptic and postsynaptic effects to inhibit the release of neurotransmitters and produce a later inhibitory postsynaptic potential, respectively (Gassmann and Bettler, 2012). It has been shown that GABBR1 controls neuronal activity to prevent overexcitation, thereby preventing excitotoxicity and cell death. Selective continuous activation of GABBR1 can provide neuroprotection *in vitro* and *in vivo* models of cerebral ischemia (Kim et al., 2014). Moreover, studies have shown that the GABBR-mediated PI3K/AKT signaling pathway can reduce oxidative stress and neuronal cell damage in rat models of Alzheimer’s disease (Sun et al., 2020). After a stroke, activation of the PI3K/AKT signaling pathway can counteract neuronal apoptosis (Lv et al., 2019). Further, we found that GPW reduced the amount of PI3K protein in cI/R mice and inhibited the phosphorylation of AKT. Our analysis showed that GPW may be mediated by GABBR1 to produce neuroprotective effects in cI/R injury by activating PI3K/AKT signaling.

It is necessary to acknowledge the limitations of this work. First, the composition and target of GPW are statistically obtained from the database, which cannot contain all the compounds and target genes. Second, the effect of GPW on cerebral ischemia-reperfusion injury needs more clinical verification. Finally, in addition to GABBR1, PTGS2, CHRM1, CHRM3, and other targets are also highly correlated. Whether other components of GPW interact with them and participate in the treatment of cerebral ischemia-reperfusion injury is still unclear, and more experiments are needed to verify it.

## 5 Conclusion

In conclusion, this study confirmed the neuroprotective effect of GPW by regulating the PI3K/AKT signaling pathway. In addition, the combination of network analysis and molecular docking predicted that GPW might target GABBR1 for the treatment of cI/R, preliminarily indicating that GPW may be a candidate herb for further research. This work provided a theoretical basis for the clinical application of GPW.

## Data Availability

The original contributions presented in the study are included in the article/Supplementary Material, further inquiries can be directed to the corresponding author.
