# Supplementary material for: Pharmacological effects and target analysis of Guipi wan in the treatment of cerebral ischemia-reperfusion injury
Source: Front Pharmacol. 2024 Mar 7;15:1346226. doi: 10.3389/fphar.2024.1346226 (PMC10955136; doi:10.3389/fphar.2024.1346226)
Supplement: Supplementary file 1 [file Table1.DOC]

Supplementary materials


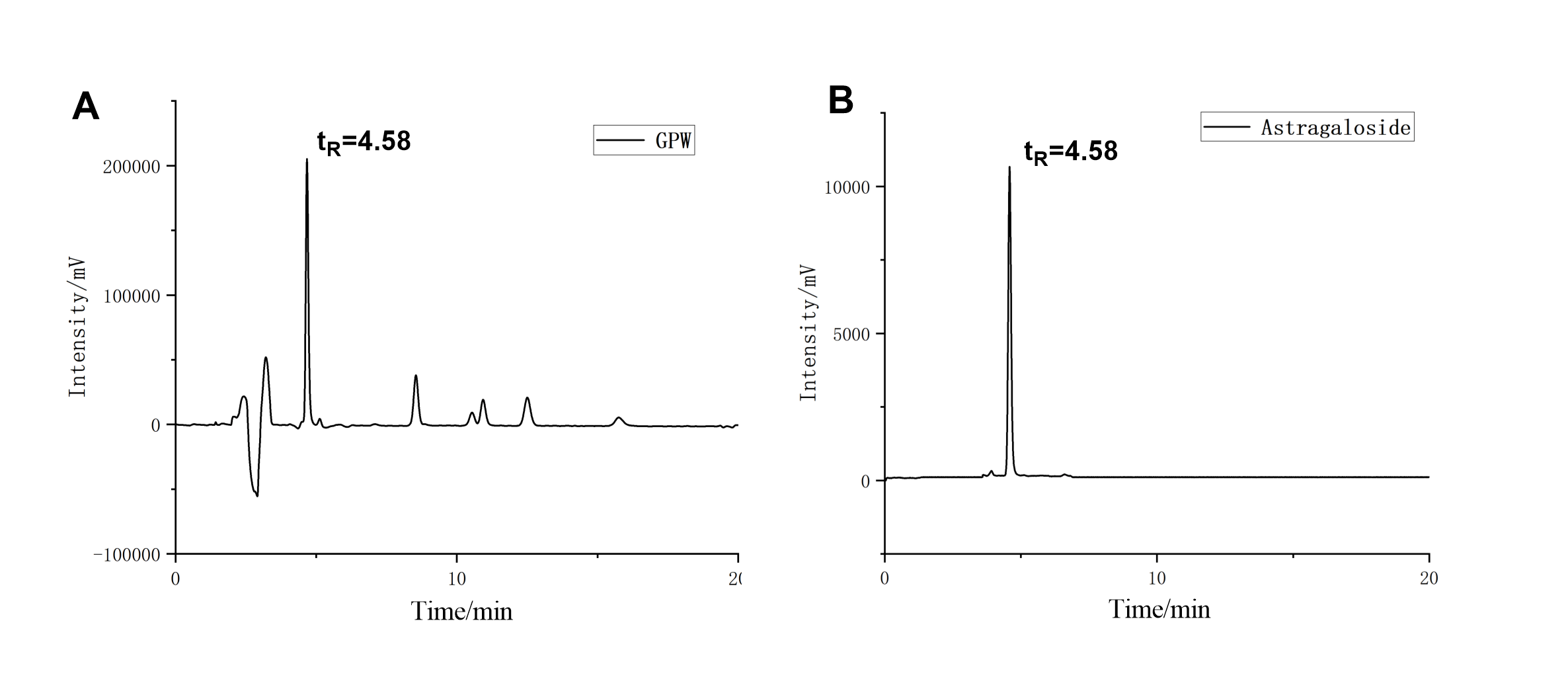


Figure S1: GPW quality standard identification (content of Astragaloside). (A) HPLC chromatogram of GPW extract (1g/mL), the peak area at t_R_=4.58 is 1707146. (B) Chromatogram of astragaloside (100μg/mL) standard, the peak area at t_R_=4.58 is 510601. The content of astragaloside in GPW was 0.33mg/mL by two-point method.


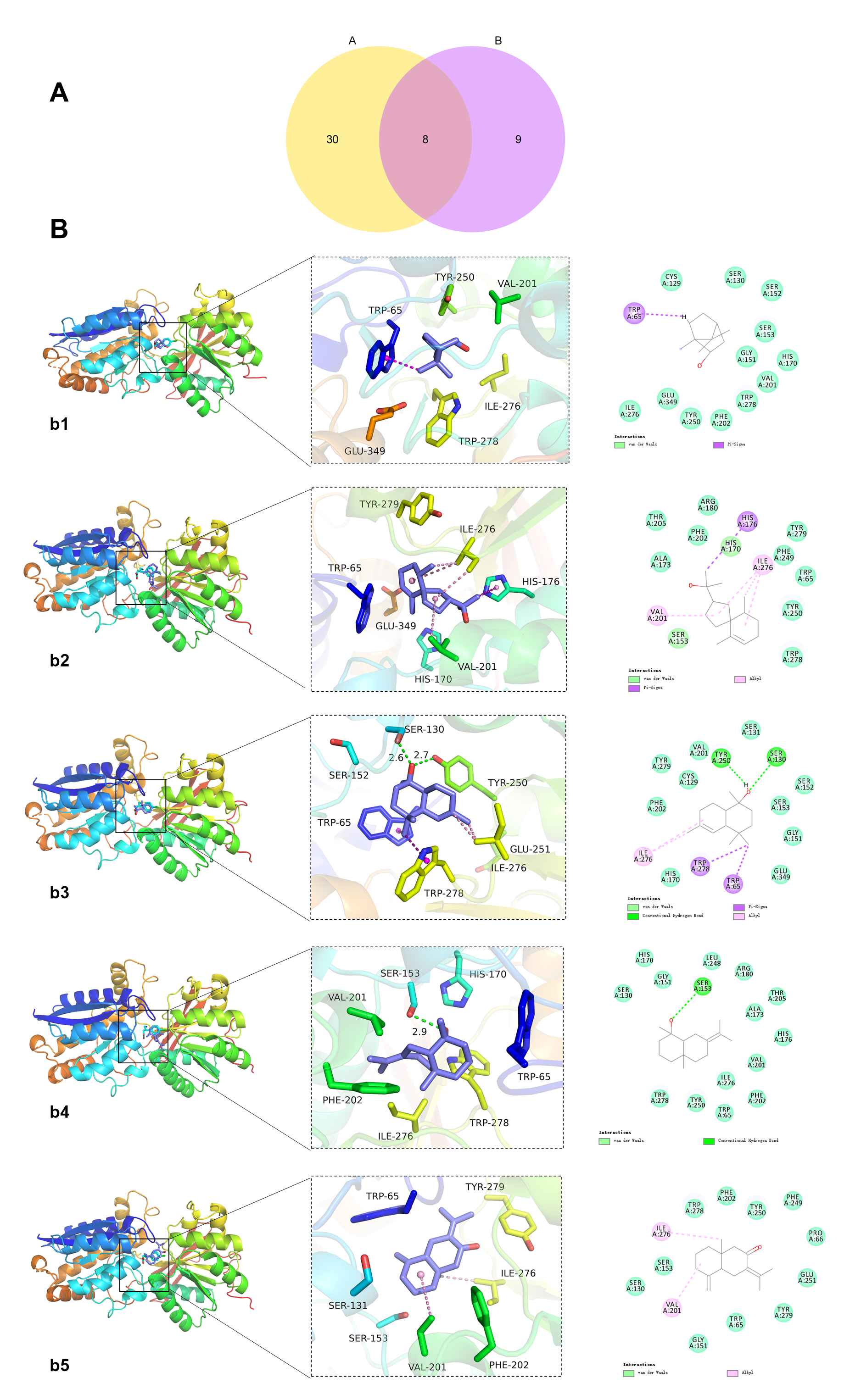


Figure S2: Molecular docking results of other 5 compounds. (A) Venn diagram of 38 components (Set A) interacting with GABBR1 and 16 primary components (Set B) preliminarily screened. (B) Results of molecular docking analysis. b1: Isoborneol; b2: 2-[(2R,5S,6S)-6,10-dimethylspiro[4.5]dec-9-en-2-yl]propan-2-ol b3: α-Cubebol; b4: Juniper camphor; b5: selina-4(14),7(11)-dien-8-one.

Table S1: Information of components of Guipi wan.

| **Number** | **Molecule name** | **Degree** |
| --- | --- | --- |
| YZ1 | S-(2-Carboxyethyl)-L-Cysteine | 88 |
| YZ2 | Tenulin | 7 |
| YZ3 | Norharman | 7 |
| YZ4 | Perlolyrine | 4 |
| YZ5 | Harmine | 13 |
| YZ6 | Harman | 9 |
| LYR1 | Thiamine | 5 |
| LYR2 | Vitamin- G | 3 |
| LYR3 | Vitamin C | 6 |
| LYR4 | NCA | 4 |
| BZ1 | (+/-)-Isoborneol | 13 |
| BZ2 | D-Camphene | 10 |
| BZ3 | Hemo-sol | 11 |
| BZ4 | α-Longipinene | 4 |
| BZ5 | (1R)-2-methyl-1-phenylprop-2-en-1-ol | 14 |
| BZ6 | (3S,8S,9S,10R,13R,14S,17R)-10,13-dimethyl-17-[(2R,5S)-5-propan-2-yloctan-2-yl]-2,3,4,7,8,9,11,12,14,15,16,17-dodecahydro-1H-cyclopenta[a]phenanthren-3-ol | 0 |
| BZ7 | Akridin | 5 |
| BZ8 | (1S,2R,4R)-Neoiso-dihydrocarveol | 5 |
| BZ9 | Atractylenolide i | 7 |
| BZ10 | Atractylenolide II | 6 |
| BZ11 | Atractylone | 19 |
| BZ12 | Juniper camphor | 10 |
| BZ13 | (5E,9Z)-3,6,10-trimethyl-4,7,8,11-tetrahydrocyclodeca[b]furan | 11 |
| BZ14 | 3β-Acetoxyatractylone | 17 |
| BZ15 | 2-[(2R,5S,6S)-6,10-dimethylspiro[4.5]dec-9-en-2-yl]propan-2-ol | 9 |
| BZ16 | Selina-4(14),7(11)-dien-8-one | 11 |
| BZ17 | Alloaromadedrene | 18 |
| BZ18 | 8β-Ethoxy atractylenolide Ⅲ | 6 |
| DS1 | Poriferasta-7,22E-dien-3beta-ol | 4 |
| S | Stigmasterol | 87 |
| DS3 | Spinasterol | 4 |
| DS4 | Stigmast-7-enol | 3 |
| DS5 | Methyl icosa-11,14-dienoate | 2 |
| DS6 | (8S,9S,10R,13R,14S,17R)-17-[(E,2R,5S)-5-ethyl-6-methylhept-3-en-2-yl]-10,13-dimethyl-1,2,4,7,8,9,11,12,14,15,16,17-dodecahydrocyclopenta[a]phenanthren-3-one | 3 |
| FL | 2-Lauroleic acid | 3 |
| DG1 | Cis-Thujopsene | 3 |
| DG3 | Stigmasterol | 6 |
| DG4 | 2,6-Di(phenyl)thiopyran-4-thione | 5 |
| S | Stigmasterol | 87 |
| A | alloaromadedrene | 12 |
| SZR1 | Daucosterol | 3 |
| SZR2 | Nuciferine | 31 |
| SZR3 | Phytosterol | 2 |
| HQ1 | (3S,8S,9S,10R,13R,14S,17R)-10,13-Dimethyl-17-[(2R,5S)-5-propan-2-yloctan-2-yl]-2,3,4,7,8,9,11,12,14,15,16,17-dodecahydro-1H-cyclopenta[a]phenanthren-3-ol | 3 |
| HQ2 | Coumarin | 14 |
| DZ1 | Sylvestrene | 3 |
| DZ2 | WLN: Q1R | 4 |
| DZ4 | Beta-carotene | 19 |
| MX1 | Benzo[a]carbazole | 16 |
| MX2 | 7-Methyl-4-(1-methylethylidene)-bicyclo[5.3.1]undec-1-en-8-ol | 2 |
| MX3 | (3aS,5R,8aS,9aS)-5,8a-dimethyl-3-methylene-5,6,7,8,9,9a-hexahydro-3aH-benzo[f]benzofuran-2-one | 6 |
| MX4 | Costuslactone | 6 |
| MX5 | Ermanthin | 9 |
| MX6 | Eudesma-5,11(13)-dien-8,12-olide | 4 |
| MX7 | Isoalantolactone | 5 |
| MX8 | 49070_FLUKA | 6 |
| MX9 | Cyperene | 5 |
| MX10 | (-)-Aromadendrene | 4 |
| MX11 | Aromadendrene oxide 2 | 5 |
| MX12 | Junipene | 7 |
| MX13 | (-)-Alpha-Longipinene | 5 |
| MX14 | (-)-Caryophyllene oxide | 11 |
| MX15 | (-)-Alpha-cedrene | 8 |
| GC1 | O-xylene | 3 |
| GC2 | M-xylene | 4 |
| GC3 | P-xylene | 9 |
| GC4 | (L)-alpha-Terpineol | 21 |
| GC5 | α-Cubebol | 13 |
| GC6 | Izoforon | 5 |
| GC7 | Isoheptane | 3 |
| GC8 | Heptan | 2 |
| GC9 | 21987_FLUKA | 3 |
| GC11 | Butylated hydroxytoluene | 20 |
| GC12 | Beta-Terpinene | 14 |
| GC13 | Anethole | 15 |
| GC15 | Octadiene | 2 |
| GC16 | Euchrenone | 10 |
| GC17 | (1S,2S)-1,2-dimethylcyclopentane | 2 |
| GC18 | 2,2-DIMETHYLPENTANE | 6 |
| GC19 | 2,3-Dimethylhexane | 2 |
| GC20 | (3S)-2,3-Dimethylpentane | 3 |
| GC21 | 5,6,7,8-Tetrahydro-2,4-dimethylquinoline | 11 |
| GC22 | (4S)-2,4-Dimethylhexane | 2 |
| GC23 | Hexa-annulenyl radical | 2 |
| GC25 | Methylcyclopentane | 2 |
| GC26 | Isohexane | 3 |
| GC27 | 3,3-Dimethylpentane | 5 |
| GC28 | 3-Methylheptane | 3 |
| GC29 | 3-Methylhexane | 3 |
| GC30 | 3-Methylpentane | 3 |
| GC31 | 3-Ethylpentane | 3 |
| GC32 | 5,6,7,8-Tetrahydro-4-methylquinoline | 8 |
| GC33 | Pentylfuran | 3 |
| GC34 | Menthol | 7 |
| GC35 | 2-Heptanone | 3 |
